# Codesigning a user-centred digital psychoeducational tool for youth mental well-being with families in Canada: study protocol for a sequential exploratory mixed methods study

**DOI:** 10.1136/bmjopen-2023-072533

**Published:** 2023-06-26

**Authors:** Stephana Julia Moss, Nicole Racine, Sofia Ahmed, Kathryn Birnie, Michal S Cherak, Janet A Curran, Donna Halperin, Scott A Halperin, Micaela Harley, Jia Hu, Laura Leppan, Angie Nickel, Kristine Russell, May Solis, Stacie Smith, Andrea Soo, Maia Stelfox, Perri R Tutelman, Henry Thomas Stelfox, Kirsten M Fiest, Jeanna Parsons Leigh

**Affiliations:** 1 Faculty of Health, Dalhousie University, Halifax, Nova Scotia, Canada; 2 School of Psychology, University of Ottawa, Ottawa, Ontario, Canada; 3 Cumming School of Medicine, University of Calgary, Calgary, Alberta, Canada; 4 School of Nursing, St Francis Xavier University, Antigonish, Nova Scotia, Canada; 5 Faculty of Medicine, Dalhousie University, Halifax, Nova Scotia, Canada

**Keywords:** MENTAL HEALTH, Public health, Child & adolescent psychiatry, Protocols & guidelines

## Abstract

**Introduction:**

On 11 March 2020, WHO declared the novel coronavirus (COVID-19) disease a global pandemic. Governments globally implemented physical distancing measures and closure of public institutions that resulted in varying implications to youth mental well-being (eg, social isolation, reduced extracurricular activities). These impacts may have detrimental short-term and long-term effects on youth mental well-being; care for youth with mental health disorders was already overstretched, underfunded and fragmented before the pandemic and youth are not often considered in mental health initiatives. There is a pressing need to partner with youth and families to target and improve youth mental well-being prior to the onset of a mental health disorder, as well as to conduct research on youth mental well-being needs related to pandemic recovery. Here we present a protocol for partnering with youth and families to codesign a user-centred digital tool for youth mental well-being.

**Methods and analysis:**

We will conduct a national research study to develop a catalogue of recommendations specific to supporting youth mental well-being, and a digital tool to support youth mental well-being through three phases of work: (1) expert consultation on data related to supporting youth mental well-being existing within our Pandemic Preparedness Research Program; (2) codesign of an innovative digital tool for youth mental well-being; and (3) assessment of the tool’s usability and acceptability.

**Ethics and dissemination:**

This study has been approved by the Dalhousie Research Ethics Board (2023-6538) and the Conjoint Health Research Ethics Board (23-0039). This study will complement ongoing foundational research in youth conducted by our team that involves partnering with youth and families to understand the unique implications of the pandemic on this population.

**STRENGTHS AND LIMITATIONS OF THIS STUDY**

- We have established a cross-national multidisciplinary team of researchers, public citizens, health professionals and knowledge users to conduct this multiphased research study.
- A catalogue of recommendations specific to supporting youth well-being and a digital tool to support youth well-being will be developed through three phases of work.
- This study will complement our ongoing foundational research in youth pandemic recovery to understand the unique implications of the pandemic on this vulnerable population.
- The primary limitations of this work are loss to follow-up and low eHealth literacy that would threaten the internal validity of our findings through selection bias; our partnerships with community-based and health system networks will help mitigate these issues.
- Missing data may also present a threat to our study through selection bias; we will pilot materials with youth and family partners on our team to ensure the length is manageable

## Background

The COVID-19 pandemic and associated public health protections have taken a devastating toll on most youth. Social isolation resulting from these measures, combined with reduced access to support services within schools and communities and fewer opportunities to engage in activities that promote general well-being, such as physical activities, extracurricular activities and socialising with friends, may have detrimental short-term and long-term effects on youth mental well-being.^1^ This is particularly evident among youth with pre-existing vulnerabilities such as exposure to familial adversity and health conditions.^2^

The widespread adoption of public health restrictions created the necessity for a rapid adaptation of existing mental health services, and in many settings, a shift towards digital health.^3^ Existing evidence suggests concerns for sustainability of digital health interventions that are not rigorously codesigned with youth and their families, which is a participatory approach to designing solutions involving stakeholders as equal collaborators^4^ that improves effectiveness of mobile health tools,^5^ as experiences and preferences are missed. Digital health interventions that only address youth with mental illness (taking on a pathologised approach) rather than acknowledging periods of poor mental well-being to address the continuum of mental well-being more broadly limit applicability and reach.^6–11^ Mental well-being support delivered virtually is an opportunity for increased access, especially for marginalised youth (eg, racialised youth) and youth living in areas with limited access to in-person services, as we recover from the COVID-19 pandemic.^12–15^

Psychoeducation is a method of knowledge translation that facilitates enhanced coping, resilience and treatment efficacy and adherence, offering a low-risk approach to delivering virtual mental well-being support.^16 17^ When youth have information about what contributes to poor mental well-being and strategies to improve it, they can implement them in their everyday lives. Care for youth with mental health disorders was overstretched and underfunded before the pandemic,^18^ and youth have reported that their opinions are ‘rarely or never’ considered in initiatives.^19^ Thus, engaging youth at the outset and establishing the goals and directions of the current project is essential to understand what support they need in the aftermath of the pandemic to target and improve youth mental well-being prior to the onset of mental health disorders. A collaborative and creative approach is needed to leverage digital mental well-being resources that youth are already using to codesign an evidence-based digital psychoeducation tool to help mitigate the negative impacts of the COVID-19 pandemic on youth well-being.

### Study aims

#### Primary aim

To codesign and pilot test a digital psychoeducational health tool—an interactive, web-based tool to help youth and their families living in Canada address poor mental well-being resulting from and persisting beyond the COVID-19 pandemic.

#### Secondary aim

To develop a catalogue of evidence-based recommendations to directly inform policies and programmes related to supporting youth and family well-being during pandemic recovery and beyond.

## Methods

### Patient and public involvement

Inclusiveness, support, cobuilding and mutual respect are the core principles that will guide this work.^20–23^ Youth and parent involvement in the current project began in 2021; they participated in group discussion alongside other stakeholders (eg, researchers, clinicians, decision makers). The research questions, protocol and this paper were jointly developed with youth and parent advisors on this team. The youth (MStelfox, SS, MH) and parent (AN, SJM, KR) partners for this project have worked with our team, framing and developing research ideas and evaluating data, and will continue to do so for this study. Youth and parent partners will be included as coauthors on resulting publications (including the current paper) and will be directly involved in dissemination through presentation of results to scientific and lay audiences. Our multidisciplinary team of youth and parent partners, researchers, clinicians and knowledge users are actively engaged in research with youth and family partners. All youth and family partners are compensated for their time.

### Phases of work and methodology

Our team is currently completing two knowledge synthesis projects (a scoping review and a systematic review) which are grounded in WHO domains of adolescent well-being (ie, health, connectedness, safety, learning, agency)^24^ to support pandemic recovery for youth and families. Our findings indicate innovative technological approaches (eg, web-based tools that provide resources, communication platforms to foster connectivity) that target the improvement of mental well-being prior to the onset of mental health disorders are a promising avenue for early prevention of costly mental health problems in an overstretched system of care. In March 2022, our team conducted a national survey of diverse youth–parent or legal guardian (hereafter referred to as parent) dyads in Canada to assess their experiences during the pandemic, including stressful events and protective mechanisms impacting well-being, their underlying drivers and associated implications and the resources accessed (including digital tools or services and perspectives on usefulness) or needed to manage individual impacts. This survey collected a large, nationally representative sample of youth–parent dyads (youth, aged 11–18 years; parents, aged 19 years or older) who provided information to be contacted for future research. We will engage this sample of representative stakeholders in a sequential exploratory mixed methods design that involves three intersecting phases of work.^25–28^

#### Phase I: knowledge synthesis and data review

##### Objectives

The primary objective of Phase I is to inform psychoeducational tool development (Phase II). The secondary objective is to provide public health and governmental policy makers with a catalogue of evidence-based recommendations to directly inform the development of policies and programmes related to supporting youth and family well-being during pandemic recovery.

##### Qualitative design

Through our national contacts and institutional affiliations we will convene a Working Group consisting of knowledge users, researchers and healthcare professionals with expertise in youth mental well-being. The Working Group will use the ‘Inform, Activate, Collaborate Framework’ developed by our team^29^ to produce a catalogue of recommendations that is informed by our team’s existing evidence base on youth well-being during pandemic recovery (2 knowledge synthesis projects (including evidence from over 20 randomised controlled trials), 1 national cross-sectional survey (1600 participants), 100 qualitative interviews with diverse (with regard to geographic location, gender, age, and diversity in household income, perceived financial difficulties, education and perceived social status of the occupation, citizenship status and potential social exclusion) youth–parent dyads (50 youth, 50 parents, interviewed separately)). Gaps in knowledge will be bridged with existing high-quality national and international evidence. Specifically, we will establish a standardised evidence-informed approach to: (1) *inform* public health and governmental decision makers of pandemic policies and stressors most impacting youth and families (eg, through the conduction of public town halls); (2) *activate* progressive youth and family participation for increased understanding and promotion of positive attitudes and behaviours to well-being (via our partnerships with key youth and family organisations); and (3) foster *collaboration* between community members, researchers, Canadian youth and family organisations and health professionals in regard to pandemic policies and impact on youth and family well-being (by tailored dissemination of our catalogue of recommendations). The goal of the catalogue of recommendations will be to inform relevant decision makers and to educate, empower and engage Canadian youth and families, including their broader communities, of the detrimental impacts of the pandemic on well-being and evidence-informed approaches, strategies and interventions to mitigate these impacts to optimise opportunities for recovery and long-term resilience. The catalogue of recommendations will be translated into English and French^30^ and openly available and accessible to a lay audience (ie, the public) on partner websites and media platforms used by youth (eg, TikTok)^31 32^ and parents (eg, Facebook), and sent directly to public health and governmental decisions makers (see letters of support).

##### Working Group process

The Working Group of youth and family partners, knowledge users, researchers and healthcare professionals with broad experience, expertise and global representation will follow four overlapping steps for knowledge synthesis and data review: (1) use established methods^33^ to gather, synthesise, critically appraise and adapt evidence into appropriate recommendations to inform tool development and broader policy initiatives, (2) engage in iterative feedback and refinement, (3) incorporate emerging research from the global community and (4) liaise with research teams outside of Canada to exchange findings. The Working Group will employ a modified Appraisal of Guidelines, Research and Evaluation II tool^34^ and tailor its approach based on identified impacts and solutions. Findings will be iteratively refined in consultation with our youth partners and funnelled into Phase II.

##### Deliverable

A catalogue of recommendations specific to support youth mental well-being, including best available evidence on factors that have mitigated negative impacts of the pandemic on youth and parents.

#### Phase II: digital tool codesign

##### Objective

To codesign a generalisable (to other contexts, environments and time periods) digital psychoeducational health tool that is targeted to improve youth mental well-being.

##### Qualitative design

We will assemble a separate, national Tool Development Team consisting of knowledge users, researchers and healthcare professionals with expertise in youth mental well-being, as well as experts in user-centred design and human–computer interaction to develop a digital psychoeducational health tool for youth mental well-being with youth and parent participants.

##### Procedures

Through a series of focus groups, we will solicit feedback from the Tool Development Team to refine the catalogue of recommendations from Phase I into a single digital health psychoeducational tool that provides resources for youth during periods of poor mental well-being.^35^ The tool will be web based, providing educational information on a broad range of mental health symptoms, self-assessments, protective measures and touch point discussion topics for parents, all accessible from drop-down menus and clickable selection prompts. We selected the focus group methodology as an effective technique for exploring impressions and experiences and the contextual factors that influence those perspectives.^36^ We will initiate recruitment by inviting engaged dyads who provided contact information for future research. Dyads (n=30; 30 youth, 30 parents) will be purposively sampled to ensure equitable representation of youth gender, youth age, and diversity in youth sexual orientation (lesbian, gay, bisexual, transgender, queer/questioning, asexual, and more (LGBTQ+)), ethnicity and parental household income. A moderator will conduct focus groups (4–6 dyads/group) using a semistructured guide to elicit feedback on tool content as per the guidelines recommended by the Agency for Healthcare Research and Quality Patient Education Materials Assessment Tool^37^ including language, functionality, relatedness and usability. Attention will also be paid to broadly assessing appeal and relevance to self-identified ethnic, gender, sexual orientation, faith and other cultural considerations. During the first focus groups, participants will see the prototype tool. In subsequent focus groups, participants will see a modified prototype based on feedback from the previous focus groups. We anticipate three cycles of development and refinement until no further modifications (optimal content and user-friendly format) are proposed (80% of human factor issues are identified after three design cycles).^38^ The Tool Development Team will evaluate feedback from focus groups, revise the tool and solicit reviews from a new set of focus group participants before approval of the final tool. In lieu of the final focus group, we may apply a more streamlined approach used by Schorr *et al* 39 in which stakeholders will evaluate and score the understandability and actionability of the tool via electronic survey.

##### Data analysis

We will use constant comparative methods to identify themes in the Tool Development Team’s comments,^40^ communicated back to all participants to confirm perspectives are adequately represented.

##### Deliverable

An evidence-based, codesigned, user-centred digital psychoeducational health tool that is ready for pilot testing.

#### Phase III: prototype pilot testing

##### Objective

To solicit feedback from our representative group of Youth and Parent Priority Partners to assess the tool’s usability, acceptability and perceived effectiveness.

##### Quantitative and qualitative design

Cross-sectional survey (priority partners) and semistructured interviews (priority partners and healthcare providers).

##### Sample and recruitment

Youth and Parent Priority Partners will participate as dyads (one parent and one youth from the same household identified in our ongoing research programme) and will be eligible if they are a youth (≥11 and ≤18 years) and parent living in the same household in Canada, are able to understand and speak English or French and are able to provide informed consent. We will initiate recruitment by inviting engaged dyads who provided contact information in our previous studies to be engaged in future research. Dyads (n=50; 50 youth, 50 parents) will be purposively sampled to ensure equitable representation of youth gender, youth age, and diversity in youth sexual orientation (LGBTQ+), ethnicity and parental household income. Additional avenues for recruitment (eg, community-based networks) will be applied to obtain the experiences of subpopulations that may be under-represented in the participant list generated from our ongoing research. Interviews will be conducted by experienced bilingual team members.

##### Procedures

Prior to user testing, participants will complete a baseline questionnaire including the WHO-Five Well-Being Index, a short self-reported measure of current mental well-being.^41^ Research staff will meet with youth and parents (separately) over a 1-hour session to complete the tool (virtually). Youth and Parent Priority Partners will be asked to perform tasks such as selecting responses from a menu of questions, navigating to specific sections and interacting with video content while verbally describing their thought process via think-aloud exercises.^42–44^ Immediately after the session we will administer an online questionnaire of the System Usability Scale, a validated 10-item questionnaire that uses a 5-point Likert scale to assess usability.^45^ We will assess acceptability with two internally generated questions (‘How acceptable did you find it to have mental wellbeing support presented in the tool?’ and ‘How acceptable did you find the tool overall?’) using a 5-point Likert scale. We will measure perceived effectiveness with four internally generated questions (‘How helpful was the tool in preparing you to communicate with your friends and family about your mental wellbeing?’, ‘How well did the tool function in helping you understand your mental wellbeing?’, ‘Do you think the tool helped you think through what were the best goals for improving your mental wellbeing?’ and ‘Would you recommend the tool to a friend if they were working through a period of poor mental wellbeing?’). The first two questions will be measured on a 5-point Likert scale and the latter two will be ‘yes’ or ‘no’ questions. Within the same session, researchers will ask open-ended questions to elicit dyads’ impressions of the tool. We will include resources within the tool for psychological and social work consultants including the clinical psychologists (NR, KB, PRT) who are part of the grant team in the event dyads develop psychological distress. We will also elicit qualitative feedback from youth mental health providers (n=30) to assess tool usability and acceptability from the providers’ perspective. Research staff will give providers an overview of the tool, show each section and explain the intended purpose of each section. Research staff will then conduct interviews to elicit feedback on usability and acceptability of the tool.

##### Data analysis

Survey responses will be summarised using medians with IQR or frequencies with proportions. We will summarise youth and parent survey responses separately and conduct subgroup analyses on variables of interest (eg, age, self-identified gender, sexual orientation, region, ethnicity, household income, perceived financial difficulties, education and perceived social status of the occupation, citizenship status and potential social exclusion) to identify associations with sociodemographic factors. We will also make paired (by dyad) comparisons for questions using Wilcoxon signed-rank tests for Likert scale questions and McNemar’s test for categorical questions. Interviews will be audio recorded, transcribed and coded for major themes using constant comparative methods.^40^ We will integrate our qualitative and quantitative findings by using qualitative results to interrogate quantitative results, aiding interpretation of heterogeneity as a rich source of information and to identify research gaps.^46^

##### Deliverable

Enriched understanding of the tool’s usability, acceptability and perceived effectiveness and relevance to key sociodemographic differences in order to optimise tailored communication of the tool.

### Feasibility and mitigating strategies

Our existing relationships, COVID-19 research infrastructure and active partnerships with national youth and family organisations, in collaboration with the Centre for Addiction and Mental Health and National Youth Advisory Council, will enable us to facilitate timely uptake and dissemination of our results. The primary limitations of this work are loss to follow-up and low eHealth literacy that would threaten the internal validity of our findings through selection bias. Our partnerships with community-based and health system networks will help mitigate this issue. In all phases of work we will provide clear verbal instructions and a slowed pace of speech that uses plain language and avoids jargon. Missing data may also present a threat to our study through selection bias. We will pilot materials with youth and family partners on our team to ensure the length is manageable (less than 20 min to complete). Finally, it is important to recognise that the COVID-19 pandemic and associated public health measures are not universally detrimental to youth; they may have experienced a reduction in school-life conflict^47^ or in stress from potential high-risk COVID-19 exposures in their classrooms.^48 49^ Our deliverables will be relevant to youth experiencing mental health difficulties in the COVID-19 recovery period and beyond.

## Supplementary Material

Reviewer comments

Author's manuscript
