# Supplementary material for: Codesigning a user-centred digital psychoeducational tool for youth mental well-being with families in Canada: study protocol for a sequential exploratory mixed methods study
Source: BMJ Open. 2023 Jun 26;13(6):e072533. doi: 10.1136/bmjopen-2023-072533 (PMC10410808; doi:10.1136/bmjopen-2023-072533)
Supplement: Author's manuscript [file bmjopen-2023-072533.draft_revisions.pdf]

BMJ Open is committed to open peer review. As part of this commitment we make the peer review history of every article we publish publicly available.

When an article is published we post the peer reviewers' comments and the authors' responses online. We also post the versions of the paper that were used during peer review. These are the versions that the peer review comments apply to.

The versions of the paper that follow are the versions that were submitted during the peer review process. They are not the versions of record or the final published versions. They should not be cited or distributed as the published version of this manuscript.

BMJ Open is an open access journal and the full, final, typeset and author-corrected version of record of the manuscript is available on our site with no access controls, subscription charges or pay-per-view fees (http://bmjopen.bmj.com).

If you have any questions on BMJ Open's open peer review process please email info.bmjopen@bmj.com

BMJ Open

Protocol for Partnering with Youth and Families to Co-design a User-centered Digital Psychoeducational Tool for Youth Mental Wellbeing

Journal:	*BMJ Open*
Manuscript ID	bmjopen-2023-072533
Article Type:	Protocol
Date Submitted by the Author:	07-Feb-2023
Complete List of Authors:	Moss, Stephana; University of Calgary Cumming School of Medicine, Critical Care Medicine<br>Racine, Nicole; University of Ottawa<br>Ahmed, Sofia; University of Calgary<br>Birnie, kathryn; University of Calgary, Department of Anesthesiology, Perioperative and Pain Medicine and Department of Community Health Sciences<br>Cherak, Michal S; Dalhousie University<br>Curran, Janet; Dalhousie University, Nursing; IWK Health Centre, Pediatrics<br>Halperin, Donna; St Francis Xavier University<br>Halperin, Scott; Dalhousie University, Canadian Center for Vaccinology<br>Harley, Micaela; Frayme<br>Hu, Jia; University of Calgary<br>Leppan, Laura; Dalhousie University<br>Nickel, Angie; Dalhousie University<br>Russell, Kristine; Dalhousie University<br>Solis, May; Dalhousie University<br>Smith, Stacie; Young Canadians Roundtable on Health<br>Soo, Andrea; University of Calgary Cumming School of Medicine, Critical Care Medicine<br>Stelfox, Maia; Dalhousie University<br>Tutelman, Perri; Dalhousie University, Department of Psychology and Neuroscience; IWK Health Centre, Centre for Pediatric Pain Research<br>Stelfox, Henry; University of Calgary, Critical Care Medicine<br>Fiest, Kirsten; University of Calgary Cumming School of Medicine, Critical Care Medicine<br>Parsons Leigh, Jeanna; Western University, 1. Department of Medicine, Schulich School of Medicine & Dentistry
Keywords:	MENTAL HEALTH, Public health < INFECTIOUS DISEASES, Child & adolescent psychiatry < PSYCHIATRY, Protocols & guidelines < HEALTH SERVICES ADMINISTRATION & MANAGEMENT

SCHOLARONE™
Manuscripts

Protocol for Partnering with Youth and Families to Co-design a User-centered Digital Psychoeducational Tool for Youth Mental Wellbeing

Stephana Julia. Moss, Nicole Racine, Sofia B Ahmed, Kathryn Birnie, Michal S Cherak, Janet A Curran, Donna Halperin, Scott A Halperin, Micaela Harley, Jia Hu, Laura Leppan, Angie Nickel, Kristine Russell, May Solis, Stacie Smith, Andrea Soo, Maia Stelfox, Perri R Tutelman, Henry T Stelfox, Kirsten M Fiest, Jeanna Parsons Leigh

Stephana Julia Moss: Faculty of Health, Dalhousie University, Halifax, NS

Nicole Racine: School of Psychology, University of Ottawa, Ottawa, Canada

Sofia Ahmed: Cumming School of Medicine, University of Calgary, Calgary, Canada

Kathryn Birnie: Department of Anesthesiology, Perioperative, and Pain Medicine; Department of Community Health Sciences, University of Calgary, Calgary, AB

Michal Cherak: Faculty of Health, Dalhousie University, Halifax, NS

Janet Curran: Quality Improvement and Patient Safety, IWK Health, Nova Scotia Health, Nova Scotia Department of Health, Halifax, Canada

Donna Halperin: Rankin School of Nursing, St. Francis Xavier University, Antigonish, NS

Scott A. Halperin: Canadian Center for Vaccinology, Dalhousie University, IWK Health and Nova Scotia Health, Halifax, Canada

Micaela Harley: Frayme, The Royal's Institute of Mental Health Research

Jia Hu: Department of Community Health Sciences, University of Calgary, Calgary AB

Laura Leppan: Faculty of Medicine, Faculty of Health, Dalhousie University, Halifax, Canada

Angie Nickel: Faculty of Health, Dalhousie University, Halifax, NS

Kristine Russell: Faculty of Health, Dalhousie University, Halifax, NS

May Solis: Faculty of Health, Dalhousie University, Halifax, NS

Stacie Smith: Young Canadian Roundtable on Health, Toronto, ON

Andrea Soo: Department of Critical Care Medicine, University of Calgary, Calgary, AB

Maia Stelfox: Faculty of Health, Dalhousie University, Halifax, NS

Perri Tutelman: Cumming School of Medicine, University of Calgary, Calgary, Canada

Henry T Stelfox: Department of Critical Care Medicine and O'Brien Institute for Public Health, University of Calgary, Calgary, AB

Kirsten Fiest: Department of Critical Care Medicine and O'Brien Institute for Public Health, University of Calgary, Calgary, AB

Jeanna Parsons Leigh: Faculty of Health, Dalhousie University, Halifax, NS

Corresponding author: Jeanna Parsons Leigh, J.ParsonsLeigh@dal.ca

For peer review only

ABSTRACT

Introduction

On March 11, 2020 the World Health Organization declared the novel coronavirus COVID-19 disease a global pandemic. Governments globally implemented physical distancing measures and closure of public institutions that resulted in varying implications to youth mental wellbeing (e.g., social isolation, reduced extra-curricular activities). These impacts may have detrimental short- and long-term effects on youth mental wellbeing; care for youth with mental health disorders was already overstretched, underfunded, and fragmented before the pandemic and youth are not often considered in mental health initiatives. There is a pressing need to partner with youth and families to target and improve youth mental wellbeing prior to the onset of a mental health disorder, as well as to conduct research on youth mental wellbeing needs related to pandemic recovery. Here we present a protocol for partnering with youth and families to co-design a user-centered digital tool for youth mental wellbeing.

Methods

We will conduct a national research study to develop a catalogue of recommendations specific to supporting youth mental wellbeing, and a digital tool to support youth mental wellbeing through three phases of work: 1) expert consultation on data related to supporting youth mental wellbeing existing within our Pandemic Preparedness research program; 2) co-design of an innovative digital tool for youth mental wellbeing; and 3) assessment of the tool's usability and acceptability. This study will complement ongoing foundational research in youth conducted by our team that involves partnering with youth and families to understand the unique implications of the pandemic on this population.

Discussion

The ultimate goal of the proposed work is to build on our foundational work by cataloguing recommendations specific to supporting youth mental wellbeing and developing a digital health tool in partnership with youth and families to support pandemic recovery and resilience in the face of emerging public health crises.

Key Words

Youth; Families; Mental Wellbeing; COVID19; Pandemic; Digital Tool; Psychoeducation

For peer review only - http://bmjopen.bmj.com/site/about/guidelines.xhtml

ARTICLE SUMMARY

Strengths and limitations of this study

- We have established a cross-national multidisciplinary team of researchers, public citizens, health professionals, and knowledge users to conduct this multi-phased research study.
- A catalogue of recommendations specific to supporting youth wellbeing and a digital tool to support youth wellbeing will be developed through three phases of work.
- This study will complement our ongoing foundational research in youth pandemic recovery to understand the unique implications of the pandemic on this vulnerable population.
- The primary limitations of this work are loss to follow-up and low eHealth literacy that would threaten the internal validity of our findings through selection bias; our partnerships with community-based and health system networks will help to mitigate these issues.
- Missing data may also present a threat to our study through selection bias; we will pilot materials with youth and family partners on our team to ensure the length is manageable

For peer review only

BACKGROUND

The COVID-19 pandemic and associated public health protections have taken a devastating toll on most youth. Social isolation resulting from these measures, combined with reduced access to support services within schools and communities and fewer opportunities to engage in activities that promote general wellbeing, such as physical activity, extra-curricular activities, and socializing with friends may have detrimental short-and long-term effects on youth mental wellbeing.[1] This is particularly evident among youth with pre-existing vulnerabilities such as exposure to familial adversity and health conditions.[2]

The widespread adoption of public health restrictions created the necessity for a rapid adaptation of existing mental health services, and in many settings, a shift towards digital health.[3] Existing evidence suggests concerns for sustainability of digital health interventions that are not rigorously co-designed with youth and their families, which is a participatory approach to designing solutions involving stakeholders as equal collaborators[4] that improves effectiveness of mobile health tools,[5] as experiences and preferences are missed. Digital health interventions that only address youth with mental illness (taking on a pathologized approach) rather than acknowledging periods of poor mental wellbeing to address the continuum of mental wellbeing more broadly limits applicability and reach.[6-11] Mental wellbeing support delivered virtually is an opportunity for increased access, especially for marginalized youth (e.g., racialized youth) and youth living in areas with limited access to in-person services, as we recover from the COVID-19 pandemic.[12-15]

Psychoeducation is a method of knowledge translation that facilitates enhanced coping, resilience, and treatment efficacy and adherence, offering a low-risk approach to delivering virtual mental wellbeing support.[16 17] When youth have information about what contributes to poor mental wellbeing and strategies to improve it, they can implement them in their everyday lives. Care for youth with mental health disorders was overstretched and underfunded before the pandemic,[18] and youth have reported that their opinions are 'rarely or never' considered in initiatives.[19] Thus, engaging youth at the outset and establishing the goals and directions of the current project is essential to understand what supports they need in the aftermath of the pandemic to target and improve youth mental wellbeing prior to the onset of mental health disorders. A collaborative and creative approach is needed to leverage digital mental wellbeing resources that youth are already using to co-design an evidence-based digital psychoeducation tool to help mitigate negative impacts of the COVID-19 pandemic on youth wellbeing.

Study aims

Primary aim

To co-design and pilot test a digital psychoeducational health tool—an interactive, web-based tool to help youth and their families living in Canada address poor mental wellbeing resulting from and persisting beyond the COVID-19 pandemic.

Secondary aim

To develop a catalogue of evidence-based recommendations to directly inform policies and programs related to supporting youth and family wellbeing during pandemic recovery and beyond.

For peer review only - http://bmjopen.bmj.com/site/about/guidelines.xhtml

Patient and public involvement

Inclusiveness, support, co-building, and mutual respect are the core principles that will guide this work.[20-23] Youth and parent involvement in the current project began in 2021; they participated in group discussion alongside other stakeholders (e.g., researchers, clinicians, decision makers). The research questions, protocol, and this paper were jointly developed with youth and parent advisors on this team. The youth (MS, SS, MH) and parent (AN, SM, KR) partners for this project have worked with our team, framing and developing research ideas and evaluating data, and will continue to do so for this study. Youth and parent partners will be included as co-authors on resulting publications (including the current paper) and will be directly involved in dissemination through presentation of results to scientific and lay audiences. Our multidisciplinary team of youth and parent partners, researchers, clinicians, and knowledge users are actively engaged in research with youth and family partners. All youth and family partners are compensated for their time.

METHODS

Phases of work and methodology

Our team is currently completing two knowledge synthesis projects (a scoping review and a systematic review) which are grounded in the World Health Organization (WHO) domains of adolescent wellbeing (i.e., health, connectedness, safety, learning, agency)[24 25] to support pandemic recovery for youth and families. Our findings indicate innovative technological approaches (e.g., web-based tools that provide resources, communication platforms to foster connectivity) that target the improvement of mental wellbeing prior to the onset of mental health disorders is a promising avenue for early prevention of costly mental health problems in an overstretched system of care. In March 2022 our team conducted a national survey of diverse youth-parent or legal guardian (hereafter referred to as parent) dyads in Canada to assess their experiences during the pandemic, including stressful events and protective mechanisms impacting wellbeing, their underlying drivers and associated implications, and the resources accessed (including digital tools or services and perspectives on usefulness) or needed to manage individual impacts. This survey collected a large, nationally representative sample of youth-parent dyads (youth, aged 11-18 years; parents, aged 19 years or older) who provided information to be contacted for future research. We will engage this sample of representative stakeholders in a sequential exploratory mixed methods design that involves three intersecting phases of work.[26-29]

Phase I: Knowledge synthesis and data review

Objectives: The primary objective of Phase I is to inform psychoeducational tool development (Phase II). The secondary objective is to provide public health and governmental policy makers with a catalogue of evidence-based recommendations to directly inform the development of policies and programs related to supporting youth and family wellbeing during pandemic recovery.

Qualitative Design: Through our national contacts and institutional affiliations we will convene a Working Group consisting of knowledge users, researchers, and healthcare professionals with expertise in youth mental wellbeing. The Working Group will utilize the "Inform, Activate, Collaborate Framework" developed by our

For peer review only - http://bmjopen.bmj.com/site/about/guidelines.xhtml

team[30] to produce a catalogue of recommendations that is informed by our team's existing evidence base on youth wellbeing during pandemic recovery (two knowledge synthesis projects (including evidence from over 20 randomized controlled trials), one national cross-sectional survey (1,600 participants), 100 qualitative interviews with diverse (with regard to geographic location, gender, age, and diversity in household income, perceived financial difficulties, education and perceived social status of the occupation, citizenship status and potential social exclusion) youth-parent dyads (50 youth, 50 parents, interviewed separately)). Gaps in knowledge will be bridged with existing high-quality national and international evidence. Specifically, we will establish a standardized evidence-informed approach to: (1) *inform* public health and governmental decision makers of pandemic policies and stressors most impacting youth and families (e.g., through the conduction of public Town Halls); (2) *activate* progressive youth and family participation for increased understanding and promotion of positive attitudes and behaviors to wellbeing (via our partnerships with key youth and family organizations); and (3) foster *collaboration* between community members, researchers, Canadian youth and family organizations, and health professionals in regards to pandemic policies and impact on youth and family wellbeing (by tailored dissemination of our catalogue of recommendations). The goal of the catalogue of recommendations will be to inform relevant decision makers and to educate, empower, and engage and Canadian youth and families, including their broader communities, of the detrimental impacts of the pandemic on wellbeing and evidence-informed approaches, strategies, and interventions to mitigate these impacts to optimize opportunities for recovery and long-term resilience. The catalogue of recommendations will be translated into English and French[31] and openly available and accessible to a lay audience (i.e., the public) on partner websites and media platforms used by youth (e.g. TikTok)[32 33] and parents (e.g. Facebook), and sent directly to public health and governmental decisions makers (see letters of support).

Working Group Process: The Working Group of youth and family partners, knowledge users, researchers, and healthcare professionals with broad experience, expertise, and global representation will follow four overlapping steps for knowledge synthesis and data review: (1) use established methods[34] to gather, synthesize, critically appraise, and adapt evidence into appropriate recommendations to inform tool development and broader policy initiatives, (2) engage in iterative feedback and refinement, (3) incorporate emerging research from the global community, and (4) liaise with research teams outside of Canada to exchange findings. The Working Group will employ a modified AGREE II tool[35] and tailor its approach based on identified impacts and solutions. Findings will be iteratively refined in consultation with our youth partners and funneled into Phase II.

Deliverable: A catalogue of recommendations specific to support youth mental wellbeing, including best available evidence on factors that have mitigated negative impacts of the pandemic on youth and parents.

Phase II: Digital tool co-design

Objective: To co-design a generalizable (to other contexts, environments, and time periods) digital psychoeducational health tool that is targeted to improve youth mental wellbeing.

Qualitative Design: We will assemble a separate, national Tool Development Team consisting of knowledge users, researchers, and healthcare professionals with expertise in youth mental wellbeing, as well as experts in

For peer review only - http://bmjopen.bmj.com/site/about/guidelines.xhtml

user-centered design and human-computer interaction to develop a digital psychoeducational health tool for youth mental wellbeing with youth and parent participants.

Procedures: Through a series of focus groups, we will solicit feedback from the Tool Development Team to refine the catalogue of recommendations from Phase I into a single digital health psychoeducational tool that provides resources for youth during periods of poor mental wellbeing.[36] The tool will be web-based, providing educational information on a broad range of mental health symptoms, self-assessments, protective measures, and touch-point discussion topics for parents, all accessible from drop-down menus and clickable selection prompts. We selected the focus group methodology as an effective technique for exploring impressions and experiences and the contextual factors that influence those perspectives.[37] We will initiate recruitment by inviting engaged dyads who provided contact information for future research. Dyads (N=30; 30 youth, 30 parents) will be purposively sampled to ensure equitable representation of youth gender, youth age, and diversity in youth sexual orientation (LGBTQ+) ethnicity and parental household income. A moderator will conduct focus groups (4-6 dyads/group) using a semi-structured guide to elicit feedback on tool content as per the guidelines recommended by the Agency for Healthcare Research and Quality Patient Education Materials Assessment Tools (PEMAT)[38] including language, functionality, relatedness and usability. Attention will also be paid to broadly assessing appeal and relevance to self-identified ethnic, gender, sexual orientation, faith, and other cultural considerations. During the first focus groups, participants will see the prototype tool. In subsequent focus groups, participants will see a modified prototype based on feedback from the previous focus groups. We anticipate 3 cycles of development and refinement until no further modifications (optimal content and user-friendly format) are proposed (80% of human factor issues are identified after 3 design cycles).[39] The Tool Development Team will evaluate feedback from focus groups, revise the tool and solicit reviews from a new set of focus group participants before approval of the final tool. In lieu of the final focus group, we may apply a more streamlined approach used by Schorr et al.[40] in which stakeholders will evaluate and score the understandability and actionability of the tool via electronic survey.

Data Analysis: We will use constant comparative methods to identify themes in the Tool Development Team's comments,[41] communicated back to all participants to confirm perspectives are adequately represented.

Deliverable: An evidence-based, co-designed, user-centered digital psychoeducational health tool that is ready for pilot testing.

<u>Phase III: Prototype pilot testing</u>

Objective: To solicit feedback from our representative group of Youth and Parent Priority Partners to assess the tool's usability, acceptability, and perceived effectiveness.

Quantitative and Qualitative Design: Cross-sectional survey (priority partners) and semi-structured interviews (priority partners and healthcare providers)

Sample & Recruitment: Youth and Parent Priority Partners will participate as dyads (1 parent, 1 youth from the same household identified in our ongoing research program) and will be eligible if they are a youth (≥11 and ≤18 years) and parent living in the same household in Canada, are able to understand and speak English or French,

For peer review only - http://bmjopen.bmj.com/site/about/guidelines.xhtml

and able to provide informed consent. We will initiate recruitment by inviting engaged dyads who provided contact information in our previous studies to be engaged in future research. Dyads (N=50; 50 youth, 50 parents) will be purposively sampled to ensure equitable representation of youth gender, youth age, and diversity in youth sexual orientation (LGBTQ+), ethnicity and parental household income. Additional avenues for recruitment (e.g., community-based networks) will be applied to obtain the experiences of sub-populations that may be underrepresented in the participant list generated from our ongoing research. Interviews will be conducted by experienced bilingual team members.

Procedures: Prior to user testing, participants will complete a baseline questionnaire including the WHO-Five Well-Being Index (WHO-5), a short self-reported measure of current mental wellbeing.[42] Research staff will meet with youth and parents (separately) over a 1-hr session to complete the tool (virtually). Youth and Parent Priority Partners will be asked to perform tasks such as selecting responses from a menu of questions, navigating to specific sections, and interacting with video content while verbally describing their thought process via think-aloud exercises.[43-45] Immediately after the session we will administer an online questionnaire of the System Usability Scale, a validated 10-item questionnaire that uses a 5-point Likert scale to assess usability.[46] We will assess acceptability with two internally generated questions ("How acceptable did you find it to have mental wellbeing support presented in the tool?" and "How acceptable did you find the tool overall?") using a 5-point Likert scale. We will measure perceived effectiveness with four internally generated questions, ("How helpful was the tool in preparing you to communicate with your friends and family about your mental wellbeing?", "How well did the tool function in helping you understand your mental wellbeing?", "Do you think the tool helped you think through what were the best goals for improving your mental wellbeing?", and "Would you recommend the tool to a friend if they were working through a period of poor mental wellbeing?"). The first two questions will be measured on a 5-point Likert scale and the latter two will be 'yes' or 'no' questions. Within the same session, researchers will ask open-ended questions to elicit dyads' impressions of the tool. We will include resources within the tool for psychological and social work consultants including the clinical psychologists (NR, KB, PT) who are part of the grant team in the event dyads develop psychological distress. We will also elicit qualitative feedback from youth mental health providers (N=30) to assess tool usability and acceptability from the providers' perspective. Research staff will give providers an overview of the tool, show each section, and explain the intended purpose of each section. Research staff will then conduct interviews to elicit feedback on usability and acceptability of the tool.

Data Analysis: Survey responses will be summarized using medians with interquartile range or frequencies with proportions. We will summarize youth and parent survey responses separately and conduct subgroup analyses on variables of interest (e.g., age, self-identified gender, sexual orientation, region, ethnicity, household income, perceived financial difficulties, education and perceived social status of the occupation, citizenship status and potential social exclusion) to identify associations with sociodemographic factors. We will also make paired (by dyad) comparisons for questions using Wilcoxon signed rank tests for Likert scale questions and McNemar's test

For peer review only - http://bmjopen.bmj.com/site/about/guidelines.xhtml

for categorical questions. Interviews will be audio-recorded, transcribed, and coded for major themes using constant comparative methods.[41] We will integrate our qualitative and quantitative findings by using qualitative results to interrogate quantitative results, aiding interpretation of heterogeneity as a rich source of information and to identify research gaps.[47]

Deliverable: Enriched understanding of the tool's usability, acceptability, and perceived effectiveness and relevance to key sociodemographic differences in order to optimize tailored communication of the tool.

Feasibility and mitigating strategies

Our existing relationships, COVID-19 research infrastructure, and active partnerships with national youth and family organizations, in collaboration with the Centre for Addiction and Mental Health and National Youth Advisory Council will enable us to facilitate timely uptake and dissemination of our results. The primary limitations of this work are loss to follow-up and low eHealth literacy that would threaten the internal validity of our findings through selection bias. Our partnerships with community-based and health system networks will help to mitigate this issue. In all phases of work we will provide clear verbal instructions and a slowed pace of speech that uses plain and avoids jargon. Missing data may also present a threat to our study through selection bias. We will pilot materials with youth and family partners on our team to ensure the length is manageable (less than. 20 minutes to complete). Finally, it is important to recognize that the COVID-19 pandemic and associated public health measures are not universally detrimental to youth; they may have experienced a reduction in school-life conflict[48] or in stress from potential high-risk COVID-19 exposures in their classrooms.[49 50] Our deliverables will be relevant to youth experiencing mental health difficulties in the COVID-19 recovery period, and beyond.

DISCUSSION

COVID-19 has taken a devastating toll on youth.[1 7 51-53] This stakeholder-informed pandemic preparedness research is grounded in meaningful community engagement with a clear focus on upstream and midstream preparedness and downstream recovery efforts that put public participation in the development of social and health equity at the forefront for the current and future infectious disease outbreaks. In collaboration with our national partners and organizational supports, this work will fill persistent knowledge gaps with public-driven recovery recommendations and interventions that address a problem of high public health importance. These recommendations and interventions are supported by extensive prior research, optimized through engagement with priority partners and scientific experts, and are designed to be readily generalizable to maximize public health impact; future work includes validating the tool in other populations of youth outside of Canada. Partnering with youth to identify and understand unique implications of the COVID-19 pandemic and co-create a multidisciplinary hub for pandemic preparedness is essential to support national public health resilience moving forward.

For peer review only - http://bmjopen.bmj.com/site/about/guidelines.xhtml

Funding Statement

Dr. Parsons Leigh obtained funding for this work from the Canadian Institutes of Health Research (CIHR) (#483889). Dr. Moss was supported by a CIHR Banting Postdoctoral Fellowship. CIHR has no role in the study design; data collection, management, analysis, or interpretation; writing of this or future reports; the decision to submit for publication; or ultimate authority over any of these activities.

Author Statement

Conceptualization: SJM, NR, HTS, JPL

Resources: HTS, KMF, JPL

Software: JPL

Supervision: NR, HTS, KMF, JPL

Writing – original draft: SJM, MSC, JPL

Writing – review and editing: ALL

Data Sharing and Availability of Materials

Not applicable. No data was analyzed for development of this protocol.

Competing interests

The authors declare that they have no competing interests relevant to this work.

Acknowledgements

The authors wish to acknowledge their organizational partners including Solutions for Kids in Pain, the O'Brien Institute for Public Health, the Maritime and Alberta SPOR SUPPORT Units, 19 to Zero, The Sandbox Project, Children's Healthcare Canada, the Centre for Addictions and Mental Health, the Centre for Child, Youth, and Family Mental Health, and the Young Canadians Roundtable on Mental Health. The authors thank the youth and family dyads who are engaged and invested in bettering tomorrow for others.

For peer review only

Bibiliography

1. Marques de Miranda D, da Silva Athanasio B, Sena Oliveira AC, et al. How is COVID-19 pandemic impacting mental health of children and adolescents? *International Journal of Disaster Risk Reduction* 2020;51:101845. doi: https://doi.org/10.1016/j.ijdrr.2020.101845
2. Silliman Cohen RI, Bosk EA. Vulnerable Youth and the COVID-19 Pandemic. *Pediatrics* 2020;146(1):e20201306. doi: 10.1542/peds.2020-1306
3. Ramalho R, Adiukwu F, Gashi Bytyçi D, et al. Telepsychiatry and healthcare access inequities during the COVID-19 pandemic. *Asian Journal of Psychiatry* 2020;53:102234. doi: https://doi.org/10.1016/j.ajp.2020.102234
4. Slattery P, Saeri AK, Bragge P. Research co-design in health: a rapid overview of reviews. *Health Research Policy and Systems* 2020;18(1):17. doi: 10.1186/s12961-020-0528-9
5. Eyles H, Jull A, Dobson R, et al. Co-design of mHealth Delivered Interventions: A Systematic Review to Assess Key Methods and Processes. *Current Nutrition Reports* 2016;5(3):160-67. doi: 10.1007/s13668-016-0165-7
6. Pellicano E, Stears M. The hidden inequalities of COVID-19. *Autism* 2020;24(6):1309-10. doi: 10.1177/1362361320927590
7. Power E, Hughes S, Cotter D, et al. Youth mental health in the time of COVID-19. *Irish Journal of Psychological Medicine* 2020;37(4):301-05. doi: 10.1017/ipm.2020.84 [published Online First: 2020/07/02]
8. Rauschenberg C, Schick A, Goetzl C, et al. Social isolation, mental health, and use of digital interventions in youth during the COVID-19 pandemic: A nationally representative survey. *European Psychiatry* 2021;64(1):e20. doi: 10.1192/j.eurpsy.2021.17 [published Online First: 2021/03/09]
9. Jiang X, Ming W-K, You JHS. The Cost-Effectiveness of Digital Health Interventions on the Management of Cardiovascular Diseases: Systematic Review. *J Med Internet Res* 2019;21(6):e13166. doi: 10.2196/13166
10. Murray E, Hekler EB, Andersson G, et al. Evaluating Digital Health Interventions: Key Questions and Approaches. *Am J Prev Med* 2016;51(5):843-51. doi: 10.1016/j.amepre.2016.06.008
11. Pretorius C, Coyle D. Young People's Use of Digital Tools to Support Their Mental Health During Covid-19 Restrictions. *Frontiers in Digital Health* 2021;3 doi: 10.3389/fdgth.2021.763876
12. Rauschenberg C, Schick A, Hirjak D, et al. Evidence Synthesis of Digital Interventions to Mitigate the Negative Impact of the COVID-19 Pandemic on Public Mental Health: Rapid Meta-review. *J Med Internet Res* 2021;23(3):e23365. doi: 10.2196/23365
13. Malboeuf-Hurtubise C, Léger-Goodes T, Mageau GA, et al. Philosophy for children and mindfulness during COVID-19: Results from a randomized cluster trial and impact on mental health in elementary school students. *Progress in Neuro-Psychopharmacology and Biological Psychiatry* 2021;107:110260. doi: https://doi.org/10.1016/j.pnpbp.2021.110260
14. Roberts LW, Chan S, Torous J. New tests, new tools: mobile and connected technologies in advancing psychiatric diagnosis. *NPJ Digit Med* 2018;1:20176. doi: 10.1038/s41746-017-0006-0 [published Online First: 20180226]
15. Clarke AM, Kuosmanen T, Barry MM. A systematic review of online youth mental health promotion and prevention interventions. *Journal of youth and adolescence* 2015;44:90-113.
16. Bäuml J, Froböse T, Kraemer S, et al. Psychoeducation: a basic psychotherapeutic intervention for patients with schizophrenia and their families. *Schizophr Bull* 2006;32 Suppl 1(Suppl 1):S1-S9. doi: 10.1093/schbul/sbl017 [published Online First: 2006/08/18]
17. Lincoln TM, Wilhelm K, Nestoriuc Y. Effectiveness of psychoeducation for relapse, symptoms, knowledge, adherence and functioning in psychotic disorders: A meta-analysis. *Schizophrenia Research* 2007;96(1):232-45. doi: https://doi.org/10.1016/j.schres.2007.07.022
18. Asmundson GJG, Blackstock C, Bourque MC, et al. Easing the disruption of COVID-19: supporting the mental health of the people of Canada—October 2020—an RSC Policy Briefing. *FACETS* 2020;5(1):1071-98. doi: 10.1139/facets-2020-0082

For peer review only - http://bmjopen.bmj.com/site/about/guidelines.xhtml

19. Seabury SA, Axeen S, Pauley G, et al. Measuring The Lifetime Costs Of Serious Mental Illness And The Mitigating Effects Of Educational Attainment. *Health Aff (Millwood)* 2019;38(4):652-59. doi: 10.1377/hlthaff.2018.05246
20. Gill M, Bagshaw SM, McKenzie E, et al. Patient and Family Member-Led Research in the Intensive Care Unit: A Novel Approach to Patient-Centered Research. *PLoS One* 2016;11(8):e0160947. doi: 10.1371/journal.pone.0160947 [published Online First: 2016/08/06]
21. Krewulak KD, Sept BG, Stelfox HT, et al. Feasibility and acceptability of family administration of delirium detection tools in the intensive care unit: a patient-oriented pilot study. *CMAJ Open* 2019;7(2):E294-e99. doi: 10.9778/cmajo.20180123 [published Online First: 2019/04/28]
22. Farrier CE, Stelfox HT, Fiest KM. In the pursuit of partnership: patient and family engagement in critical care medicine. *Curr Opin Crit Care* 2019;25(5):505-10. doi: 10.1097/mcc.0000000000000643 [published Online First: 2019/07/18]
23. de Grood C, Leigh JP, Bagshaw SM, et al. Patient, family and provider experiences with transfers from intensive care unit to hospital ward: a multicentre qualitative study. *CMAJ* 2018;190(22):E669-E76.
24. Ross DA, Hinton R, Melles-Brewer M, et al. Adolescent Well-Being: A Definition and Conceptual Framework. *J Adolesc Health* 2020;67(4):472-76. doi: 10.1016/j.jadohealth.2020.06.042 [published Online First: 2020/08/18]
25. Ross DA, Hinton R, Melles-Brewer M, et al. Adolescent Well-Being: A Definition and Conceptual Framework. *The Journal of adolescent health : official publication of the Society for Adolescent Medicine* 2020;67(4):472-76. doi: 10.1016/j.jadohealth.2020.06.042 [published Online First: 2020/08/13]
26. Schoonenboom J, Johnson RB. How to Construct a Mixed Methods Research Design. *Kolner Z Soz Sozpsychol* 2017;69(Suppl 2):107-31.
27. Fetters MD, Curry LA, Creswell JW. Achieving integration in mixed methods designs-principles and practices. *Health Serv Res* 2013;48(6 Pt 2):2134-56. doi: 10.1111/1475-6773.12117 [published Online First: 20131023]
28. Johnson RB, Onwuegbuzie AJ. Mixed methods research: A research paradigm whose time has come. *Educational researcher* 2004;33(7):14-26.
29. Johnson RB, Onwuegbuzie AJ, Turner LA. Toward a definition of mixed methods research. *Journal of mixed methods research* 2007;1(2):112-33.
30. Fiest KM, McIntosh CJ, Demiantschuk D, et al. Translating evidence to patient care through caregivers: a systematic review of caregiver-mediated interventions. *BMC Med* 2018;16(1):105. doi: 10.1186/s12916-018-1097-4
31. Government of Canada. Top 5 languages spoken in Canada Ottawa, ON2018 [updated Sept 18, 2020. Available from: https://www.clo-ocol.gc.ca/en/newsletter/2018/top-5-languages-spoken-canada accessed April 8 2021.
32. Thompson V. Why are youth flocking to TikTok to cope with the COVID-19 pandemic? 2020. https://news.yorku.ca/2020/04/02/why-are-youth-flocking-to-tiktok-to-cope-with-the-covid-19-pandemic/.
33. Bryson A. In age of coronavirus, could social media be kids' saving grace? *Desert Sun* 2020 April 12, 2020. https://www.desertsun.com/story/news/education/2020/04/12/coronavirus-kids-cope-pandemic-snapchat-tiktok-instagram/5126302002/ (accessed July 22, 2021).
34. Joanna Briggs Institute. Checklist for Qualitative Research: Joanna Briggs Institute; 2017 [Available from: https://jbi.global/sites/default/files/2019-05/JBI_Critical_Appraisal-Checklist_for_Qualitative_Research2017_0.pdf accessed March 23 2020.
35. Brouwers MC, Kerkvliet K, Spithoff K. The AGREE Reporting Checklist: a tool to improve reporting of clinical practice guidelines. *BMJ* 2016;352:i1152. doi: 10.1136/bmj.i1152
36. Morgan D, Krueger R, King J. Focus group kit. Thousand Oaks, CA: SAGE Publications 1998.
37. Parsons M, Greenwood J. A guide to the use of focus groups in health care research: Part 1. *Contemp Nurse* 2000;9(2):169-80. doi: 10.5172/conu.2000.9.2.169 [published Online First: 2002/02/22]
38. Shoemaker SJ, Wolf MS, Brach C. The Patient Education Materials Assessment Tool (PEMAT) and User's Guide Rockville, MD: Agency for Healthcare Research and Quality; [updated November 2020. Available from: https://www.ahrq.gov/health-literacy/patient-education/pemat.html.

For peer review only - http://bmjopen.bmj.com/site/about/guidelines.xhtml

39. Nielsen J. Usability engineering. Boston: Academic Press 1993.
40. Schorr C, Hunter K, Zuzelo PR. Understandability and Actionability of the CDC'S Printable Sepsis Patient Education Material. *Am J Crit Care* 2018;27(5):418-27. doi: 10.4037/ajcc2018121 [published Online First: 2018/09/03]
41. Sandelowski M, Barroso J, Voils CI. Using qualitative metasummary to synthesize qualitative and quantitative descriptive findings. *Res Nurs Health* 2007;30(1):99-111. doi: 10.1002/nur.20176
42. Topp CW, Østergaard SD, Søndergaard S, et al. The WHO-5 Well-Being Index: a systematic review of the literature. *Psychother Psychosom* 2015;84(3):167-76. doi: 10.1159/000376585 [published Online First: 20150328]
43. Smith JG, Alamiri NS, Biegger G, et al. Think-Aloud Testing of a Novel Safer Drinking App for College Students During COVID-19: Usability Study. *JMIR Form Res* 2022;6(2):e32716. doi: 10.2196/32716 [published Online First: 20220217]
44. Giang WCW, Bland E, Chen J, et al. User Interactions With Health Insurance Decision Aids: User Study With Retrospective Think-Aloud Interviews. *JMIR Hum Factors* 2021;8(4):e27628. doi: 10.2196/27628 [published Online First: 20211026]
45. Busse TS, Jux C, Kernebeck S, et al. Participatory Design of an Electronic Cross-Facility Health Record (ECHR) System for Pediatric Palliative Care: A Think-Aloud Study. *Children (Basel)* 2021;8(10) doi: 10.3390/children8100839 [published Online First: 20210924]
46. Bangor A, Kortum PT, Miller JT. An Empirical Evaluation of the System Usability Scale. *International Journal of Human–Computer Interaction* 2008;24(6):574-94. doi: 10.1080/10447310802205776
47. Petticrew M, Rehfuess E, Noyes J, et al. Synthesizing evidence on complex interventions: how meta-analytical, qualitative, and mixed-method approaches can contribute. *J Clin Epidemiol* 2013;66(11):1230-43. doi: 10.1016/j.jclinepi.2013.06.005 [published Online First: 2013/08/21]
48. Graham M, Weale V, Lambert KA, et al. Working at Home: The Impacts of COVID 19 on Health, Family-Work-Life Conflict, Gender, and Parental Responsibilities. *J Occup Environ Med* 2021;63(11):938-43. doi: 10.1097/jom.0000000000002337
49. Schwartz KD, Exner-Cortens D, McMorris CA, et al. COVID-19 and Student Well-Being: Stress and Mental Health during Return-to-School. *Canadian journal of school psychology* 2021;36(2):166-85. doi: https://dx.doi.org/10.1177/08295735211001653
50. Lan F-Y, Suharlim C, Kales SN, et al. Association between SARS-CoV-2 infection, exposure risk and mental health among a cohort of essential retail workers in the USA. *Occupational and Environmental Medicine* 2021;78(4):237. doi: 10.1136/oemed-2020-106774
51. Liang L, Ren H, Cao R, et al. The Effect of COVID-19 on Youth Mental Health. *Psychiatric Quarterly* 2020;91(3):841-52. doi: 10.1007/s11126-020-09744-3
52. Detsky AS, Bogoch II. COVID-19 in Canada: Experience and Response. *JAMA* 2020;324(8):743-44. doi: 10.1001/jama.2020.14033
53. Chimmula VKR, Zhang L. Time series forecasting of COVID-19 transmission in Canada using LSTM networks. *Chaos, Solitons & Fractals* 2020;135:109864. doi: https://doi.org/10.1016/j.chaos.2020.109864

For peer review only

BMJ Open

Co-designing a User-centered Digital Psychoeducational Tool for Youth Mental Wellbeing with Families in Canada: Study Protocol for a Sequential Exploratory Mixed Methods Study

Journal:	*BMJ Open*
Manuscript ID	bmjopen-2023-072533.R1
Article Type:	Protocol
Date Submitted by the Author:	02-Jun-2023
Complete List of Authors:	Moss, Stephana; University of Calgary Cumming School of Medicine, Critical Care Medicine<br>Racine, Nicole; University of Ottawa<br>Ahmed, Sofia; University of Calgary<br>Birnie, kathryn; University of Calgary, Department of Anesthesiology, Perioperative and Pain Medicine and Department of Community Health Sciences<br>Cherak, Michal S; Dalhousie University<br>Curran, Janet; Dalhousie University, Nursing; IWK Health Centre, Pediatrics<br>Halperin, Donna; St Francis Xavier University<br>Halperin, Scott; Dalhousie University, Canadian Center for Vaccinology<br>Harley, Micaela; Frayme<br>Hu, Jia; University of Calgary<br>Leppan, Laura; Dalhousie University<br>Nickel, Angie; Dalhousie University<br>Russell, Kristine; Dalhousie University<br>Solis, May; Dalhousie University<br>Smith, Stacie; Young Canadians Roundtable on Health<br>Soo, Andrea; University of Calgary Cumming School of Medicine, Critical Care Medicine<br>Stelfox, Maia; Dalhousie University<br>Tutelman, Perri; Dalhousie University, Department of Psychology and Neuroscience; IWK Health Centre, Centre for Pediatric Pain Research<br>Stelfox, Henry; University of Calgary, Critical Care Medicine<br>Fiest, Kirsten; University of Calgary Cumming School of Medicine, Critical Care Medicine<br>Parsons Leigh, Jeanna; Western University, 1. Department of Medicine, Schulich School of Medicine & Dentistry
**Primary Subject Heading**:	Public health
Secondary Subject Heading:	General practice / Family practice, Global health, Health services research, Paediatrics
Keywords:	MENTAL HEALTH, Public health < INFECTIOUS DISEASES, Child & adolescent psychiatry < PSYCHIATRY, Protocols & guidelines < HEALTH

	SERVICES ADMINISTRATION & MANAGEMENT

SCHOLARONE™
Manuscripts

Co-designing a User-centered Digital Psychoeducational Tool for Youth Mental Wellbeing with Families in Canada: Study Protocol for a Sequential Exploratory Mixed Methods Study

Stephana Julia. Moss, Nicole Racine, Sofia B Ahmed, Kathryn Birnie, Michal S Cherak, Janet A Curran, Donna Halperin, Scott A Halperin, Micaela Harley, Jia Hu, Laura Leppan, Angie Nickel, Kristine Russell, May Solis, Stacie Smith, Andrea Soo, Maia Stelfox, Perri R Tutelman, Henry T Stelfox, Kirsten M Fiest, Jeanna Parsons Leigh

Stephana Julia Moss: Faculty of Health, Dalhousie University, Halifax, NS
Nicole Racine: School of Psychology, University of Ottawa, Ottawa, Canada
Sofia Ahmed: Cumming School of Medicine, University of Calgary, Calgary, Canada
Kathryn Birnie: Department of Anesthesiology, Perioperative, and Pain Medicine; Department of Community Health Sciences, University of Calgary, Calgary, AB
Michal Cherak: Faculty of Health, Dalhousie University, Halifax, NS
Janet Curran: Quality Improvement and Patient Safety, IWK Health, Nova Scotia Health, Nova Scotia Department of Health, Halifax, Canada
Donna Halperin: Rankin School of Nursing, St. Francis Xavier University, Antigonish, NS
Scott A. Halperin: Canadian Center for Vaccinology, Dalhousie University, IWK Health and Nova Scotia Health, Halifax, Canada
Micaela Harley: Frayme, The Royal's Institute of Mental Health Research
Jia Hu: Department of Community Health Sciences, University of Calgary, Calgary AB
Laura Leppan: Faculty of Medicine, Faculty of Health, Dalhousie University, Halifax, Canada
Angie Nickel: Faculty of Health, Dalhousie University, Halifax, NS
Kristine Russell: Faculty of Health, Dalhousie University, Halifax, NS
May Solis: Faculty of Health, Dalhousie University, Halifax, NS
Stacie Smith: Young Canadian Roundtable on Health, Toronto, ON
Andrea Soo: Department of Critical Care Medicine, University of Calgary, Calgary, AB
Maia Stelfox: Faculty of Health, Dalhousie University, Halifax, NS
Perri Tutelman: Cumming School of Medicine, University of Calgary, Calgary, Canada
Henry T Stelfox: Department of Critical Care Medicine and O'Brien Institute for Public Health, University of Calgary, Calgary, AB
Kirsten Fiest: Department of Critical Care Medicine and O'Brien Institute for Public Health, University of Calgary, Calgary, AB
Jeanna Parsons Leigh: Faculty of Health, Dalhousie University, Halifax, NS
Corresponding author: Jeanna Parsons Leigh, J.ParsonsLeigh@dal.ca

For peer review only - http://bmjopen.bmj.com/site/about/guidelines.xhtml

ABSTRACT

Introduction

On March 11, 2020 the World Health Organization declared the novel coronavirus COVID-19 disease a global pandemic. Governments globally implemented physical distancing measures and closure of public institutions that resulted in varying implications to youth mental wellbeing (e.g., social isolation, reduced extra-curricular activities). These impacts may have detrimental short- and long-term effects on youth mental wellbeing; care for youth with mental health disorders was already overstretched, underfunded, and fragmented before the pandemic and youth are not often considered in mental health initiatives. There is a pressing need to partner with youth and families to target and improve youth mental wellbeing prior to the onset of a mental health disorder, as well as to conduct research on youth mental wellbeing needs related to pandemic recovery. Here we present a protocol for partnering with youth and families to co-design a user-centered digital tool for youth mental wellbeing.

Methods and analysis

We will conduct a national research study to develop a catalogue of recommendations specific to supporting youth mental wellbeing, and a digital tool to support youth mental wellbeing through three phases of work: 1) expert consultation on data related to supporting youth mental wellbeing existing within our Pandemic Preparedness research program; 2) co-design of an innovative digital tool for youth mental wellbeing; and 3) assessment of the tool's usability and acceptability.

Ethics and dissemination

This study has been approved by the Dalhousie Research Ethics Board (2023-6538) and the Conjoint Health Research Ethics Board (23-0039). This study will complement ongoing foundational research in youth conducted by our team that involves partnering with youth and families to understand the unique implications of the pandemic on this population.

Key Words

Youth; Families; Mental Wellbeing; COVID19; Pandemic; Digital Tool; Psychoeducation

For peer review only

ARTICLE SUMMARY

Strengths and limitations of this study

- We have established a cross-national multidisciplinary team of researchers, public citizens, health professionals, and knowledge users to conduct this multi-phased research study.
- A catalogue of recommendations specific to supporting youth wellbeing and a digital tool to support youth wellbeing will be developed through three phases of work.
- This study will complement our ongoing foundational research in youth pandemic recovery to understand the unique implications of the pandemic on this vulnerable population.
- The primary limitations of this work are loss to follow-up and low eHealth literacy that would threaten the internal validity of our findings through selection bias; our partnerships with community-based and health system networks will help to mitigate these issues.
- Missing data may also present a threat to our study through selection bias; we will pilot materials with youth and family partners on our team to ensure the length is manageable

For peer review only

For peer review only - http://bmjopen.bmj.com/site/about/guidelines.xhtml

BACKGROUND

The COVID-19 pandemic and associated public health protections have taken a devastating toll on most youth. Social isolation resulting from these measures, combined with reduced access to support services within schools and communities and fewer opportunities to engage in activities that promote general wellbeing, such as physical activity, extra-curricular activities, and socializing with friends may have detrimental short-and long-term effects on youth mental wellbeing.[1] This is particularly evident among youth with pre-existing vulnerabilities such as exposure to familial adversity and health conditions.[2]

The widespread adoption of public health restrictions created the necessity for a rapid adaptation of existing mental health services, and in many settings, a shift towards digital health.[3] Existing evidence suggests concerns for sustainability of digital health interventions that are not rigorously co-designed with youth and their families, which is a participatory approach to designing solutions involving stakeholders as equal collaborators[4] that improves effectiveness of mobile health tools,[5] as experiences and preferences are missed. Digital health interventions that only address youth with mental illness (taking on a pathologized approach) rather than acknowledging periods of poor mental wellbeing to address the continuum of mental wellbeing more broadly limits applicability and reach.[6-11] Mental wellbeing support delivered virtually is an opportunity for increased access, especially for marginalized youth (e.g., racialized youth) and youth living in areas with limited access to in-person services, as we recover from the COVID-19 pandemic.[12-15]

Psychoeducation is a method of knowledge translation that facilitates enhanced coping, resilience, and treatment efficacy and adherence, offering a low-risk approach to delivering virtual mental wellbeing support.[16 17] When youth have information about what contributes to poor mental wellbeing and strategies to improve it, they can implement them in their everyday lives. Care for youth with mental health disorders was overstretched and underfunded before the pandemic,[18] and youth have reported that their opinions are 'rarely or never' considered in initiatives.[19] Thus, engaging youth at the outset and establishing the goals and directions of the current project is essential to understand what supports they need in the aftermath of the pandemic to target and improve youth mental wellbeing prior to the onset of mental health disorders. A collaborative and creative approach is needed to leverage digital mental wellbeing resources that youth are already using to co-design an evidence-based digital psychoeducation tool to help mitigate negative impacts of the COVID-19 pandemic on youth wellbeing.

Study aims

Primary aim

To co-design and pilot test a digital psychoeducational health tool—an interactive, web-based tool to help youth and their families living in Canada address poor mental wellbeing resulting from and persisting beyond the COVID-19 pandemic.

Secondary aim

To develop a catalogue of evidence-based recommendations to directly inform policies and programs related to supporting youth and family wellbeing during pandemic recovery and beyond.

For peer review only - http://bmjopen.bmj.com/site/about/guidelines.xhtml

METHODS

Patient and public involvement

Inclusiveness, support, co-building, and mutual respect are the core principles that will guide this work.[20-23] Youth and parent involvement in the current project began in 2021; they participated in group discussion alongside other stakeholders (e.g., researchers, clinicians, decision makers). The research questions, protocol, and this paper were jointly developed with youth and parent advisors on this team. The youth (MS, SS, MH) and parent (AN, SM, KR) partners for this project have worked with our team, framing and developing research ideas and evaluating data, and will continue to do so for this study. Youth and parent partners will be included as co-authors on resulting publications (including the current paper) and will be directly involved in dissemination through presentation of results to scientific and lay audiences. Our multidisciplinary team of youth and parent partners, researchers, clinicians, and knowledge users are actively engaged in research with youth and family partners. All youth and family partners are compensated for their time.

Phases of work and methodology

Our team is currently completing two knowledge synthesis projects (a scoping review and a systematic review) which are grounded in the World Health Organization (WHO) domains of adolescent wellbeing (i.e., health, connectedness, safety, learning, agency)[24 25] to support pandemic recovery for youth and families. Our findings indicate innovative technological approaches (e.g., web-based tools that provide resources, communication platforms to foster connectivity) that target the improvement of mental wellbeing prior to the onset of mental health disorders is a promising avenue for early prevention of costly mental health problems in an overstretched system of care. In March 2022 our team conducted a national survey of diverse youth-parent or legal guardian (hereafter referred to as parent) dyads in Canada to assess their experiences during the pandemic, including stressful events and protective mechanisms impacting wellbeing, their underlying drivers and associated implications, and the resources accessed (including digital tools or services and perspectives on usefulness) or needed to manage individual impacts. This survey collected a large, nationally representative sample of youth-parent dyads (youth, aged 11-18 years; parents, aged 19 years or older) who provided information to be contacted for future research. We will engage this sample of representative stakeholders in a sequential exploratory mixed methods design that involves three intersecting phases of work.[26-29]

Phase I: Knowledge synthesis and data review

Objectives: The primary objective of Phase I is to inform psychoeducational tool development (Phase II). The secondary objective is to provide public health and governmental policy makers with a catalogue of evidence-based recommendations to directly inform the development of policies and programs related to supporting youth and family wellbeing during pandemic recovery.

Qualitative Design: Through our national contacts and institutional affiliations we will convene a Working Group consisting of knowledge users, researchers, and healthcare professionals with expertise in youth mental wellbeing. The Working Group will utilize the “Inform, Activate, Collaborate Framework” developed by our

For peer review only - http://bmjopen.bmj.com/site/about/guidelines.xhtml

team[30] to produce a catalogue of recommendations that is informed by our team's existing evidence base on youth wellbeing during pandemic recovery (two knowledge synthesis projects (including evidence from over 20 randomized controlled trials), one national cross-sectional survey (1,600 participants), 100 qualitative interviews with diverse (with regard to geographic location, gender, age, and diversity in household income, perceived financial difficulties, education and perceived social status of the occupation, citizenship status and potential social exclusion) youth-parent dyads (50 youth, 50 parents, interviewed separately)). Gaps in knowledge will be bridged with existing high-quality national and international evidence. Specifically, we will establish a standardized evidence-informed approach to: (1) *inform* public health and governmental decision makers of pandemic policies and stressors most impacting youth and families (e.g., through the conduction of public Town Halls); (2) *activate* progressive youth and family participation for increased understanding and promotion of positive attitudes and behaviors to wellbeing (via our partnerships with key youth and family organizations); and (3) foster *collaboration* between community members, researchers, Canadian youth and family organizations, and health professionals in regards to pandemic policies and impact on youth and family wellbeing (by tailored dissemination of our catalogue of recommendations). The goal of the catalogue of recommendations will be to inform relevant decision makers and to educate, empower, and engage and Canadian youth and families, including their broader communities, of the detrimental impacts of the pandemic on wellbeing and evidence-informed approaches, strategies, and interventions to mitigate these impacts to optimize opportunities for recovery and long-term resilience. The catalogue of recommendations will be translated into English and French[31] and openly available and accessible to a lay audience (i.e., the public) on partner websites and media platforms used by youth (e.g. TikTok)[32 33] and parents (e.g. Facebook), and sent directly to public health and governmental decisions makers (see letters of support).

Working Group Process: The Working Group of youth and family partners, knowledge users, researchers, and healthcare professionals with broad experience, expertise, and global representation will follow four overlapping steps for knowledge synthesis and data review: (1) use established methods[34] to gather, synthesize, critically appraise, and adapt evidence into appropriate recommendations to inform tool development and broader policy initiatives, (2) engage in iterative feedback and refinement, (3) incorporate emerging research from the global community, and (4) liaise with research teams outside of Canada to exchange findings. The Working Group will employ a modified AGREE II tool[35] and tailor its approach based on identified impacts and solutions. Findings will be iteratively refined in consultation with our youth partners and funneled into Phase II.

Deliverable: A catalogue of recommendations specific to support youth mental wellbeing, including best available evidence on factors that have mitigated negative impacts of the pandemic on youth and parents.

Phase II: Digital tool co-design

Objective: To co-design a generalizable (to other contexts, environments, and time periods) digital psychoeducational health tool that is targeted to improve youth mental wellbeing.

Qualitative Design: We will assemble a separate, national Tool Development Team consisting of knowledge users, researchers, and healthcare professionals with expertise in youth mental wellbeing, as well as experts in

For peer review only - http://bmjopen.bmj.com/site/about/guidelines.xhtml

user-centered design and human-computer interaction to develop a digital psychoeducational health tool for youth mental wellbeing with youth and parent participants.

Procedures: Through a series of focus groups, we will solicit feedback from the Tool Development Team to refine the catalogue of recommendations from Phase I into a single digital health psychoeducational tool that provides resources for youth during periods of poor mental wellbeing.[36] The tool will be web-based, providing educational information on a broad range of mental health symptoms, self-assessments, protective measures, and touch-point discussion topics for parents, all accessible from drop-down menus and clickable selection prompts. We selected the focus group methodology as an effective technique for exploring impressions and experiences and the contextual factors that influence those perspectives.[37] We will initiate recruitment by inviting engaged dyads who provided contact information for future research. Dyads (N=30; 30 youth, 30 parents) will be purposively sampled to ensure equitable representation of youth gender, youth age, and diversity in youth sexual orientation (LGBTQ+) ethnicity and parental household income. A moderator will conduct focus groups (4-6 dyads/group) using a semi-structured guide to elicit feedback on tool content as per the guidelines recommended by the Agency for Healthcare Research and Quality Patient Education Materials Assessment Tools (PEMAT)[38] including language, functionality, relatedness and usability. Attention will also be paid to broadly assessing appeal and relevance to self-identified ethnic, gender, sexual orientation, faith, and other cultural considerations. During the first focus groups, participants will see the prototype tool. In subsequent focus groups, participants will see a modified prototype based on feedback from the previous focus groups. We anticipate 3 cycles of development and refinement until no further modifications (optimal content and user-friendly format) are proposed (80% of human factor issues are identified after 3 design cycles).[39] The Tool Development Team will evaluate feedback from focus groups, revise the tool and solicit reviews from a new set of focus group participants before approval of the final tool. In lieu of the final focus group, we may apply a more streamlined approach used by Schorr et al.[40] in which stakeholders will evaluate and score the understandability and actionability of the tool via electronic survey.

Data Analysis: We will use constant comparative methods to identify themes in the Tool Development Team's comments,[41] communicated back to all participants to confirm perspectives are adequately represented.

Deliverable: An evidence-based, co-designed, user-centered digital psychoeducational health tool that is ready for pilot testing.

<u>Phase III: Prototype pilot testing</u>

Objective: To solicit feedback from our representative group of Youth and Parent Priority Partners to assess the tool's usability, acceptability, and perceived effectiveness.

Quantitative and Qualitative Design: Cross-sectional survey (priority partners) and semi-structured interviews (priority partners and healthcare providers)

Sample & Recruitment: Youth and Parent Priority Partners will participate as dyads (1 parent, 1 youth from the same household identified in our ongoing research program) and will be eligible if they are a youth (≥11 and ≤18 years) and parent living in the same household in Canada, are able to understand and speak English or French,

For peer review only - http://bmjopen.bmj.com/site/about/guidelines.xhtml

and able to provide informed consent. We will initiate recruitment by inviting engaged dyads who provided contact information in our previous studies to be engaged in future research. Dyads (N=50; 50 youth, 50 parents) will be purposively sampled to ensure equitable representation of youth gender, youth age, and diversity in youth sexual orientation (LGBTQ+), ethnicity and parental household income. Additional avenues for recruitment (e.g., community-based networks) will be applied to obtain the experiences of sub-populations that may be underrepresented in the participant list generated from our ongoing research. Interviews will be conducted by experienced bilingual team members.

Procedures: Prior to user testing, participants will complete a baseline questionnaire including the WHO-Five Well-Being Index (WHO-5), a short self-reported measure of current mental wellbeing.[42] Research staff will meet with youth and parents (separately) over a 1-hr session to complete the tool (virtually). Youth and Parent Priority Partners will be asked to perform tasks such as selecting responses from a menu of questions, navigating to specific sections, and interacting with video content while verbally describing their thought process via think-aloud exercises.[43-45] Immediately after the session we will administer an online questionnaire of the System Usability Scale, a validated 10-item questionnaire that uses a 5-point Likert scale to assess usability.[46] We will assess acceptability with two internally generated questions ("How acceptable did you find it to have mental wellbeing support presented in the tool?" and "How acceptable did you find the tool overall?") using a 5-point Likert scale. We will measure perceived effectiveness with four internally generated questions, ("How helpful was the tool in preparing you to communicate with your friends and family about your mental wellbeing?", "How well did the tool function in helping you understand your mental wellbeing?", "Do you think the tool helped you think through what were the best goals for improving your mental wellbeing?", and "Would you recommend the tool to a friend if they were working through a period of poor mental wellbeing?"). The first two questions will be measured on a 5-point Likert scale and the latter two will be 'yes' or 'no' questions. Within the same session, researchers will ask open-ended questions to elicit dyads' impressions of the tool. We will include resources within the tool for psychological and social work consultants including the clinical psychologists (NR, KB, PT) who are part of the grant team in the event dyads develop psychological distress. We will also elicit qualitative feedback from youth mental health providers (N=30) to assess tool usability and acceptability from the providers' perspective. Research staff will give providers an overview of the tool, show each section, and explain the intended purpose of each section. Research staff will then conduct interviews to elicit feedback on usability and acceptability of the tool.

Data Analysis: Survey responses will be summarized using medians with interquartile range or frequencies with proportions. We will summarize youth and parent survey responses separately and conduct subgroup analyses on variables of interest (e.g., age, self-identified gender, sexual orientation, region, ethnicity, household income, perceived financial difficulties, education and perceived social status of the occupation, citizenship status and potential social exclusion) to identify associations with sociodemographic factors. We will also make paired (by dyad) comparisons for questions using Wilcoxon signed rank tests for Likert scale questions and McNemar's test

For peer review only - http://bmjopen.bmj.com/site/about/guidelines.xhtml

for categorical questions. Interviews will be audio-recorded, transcribed, and coded for major themes using constant comparative methods.[41] We will integrate our qualitative and quantitative findings by using qualitative results to interrogate quantitative results, aiding interpretation of heterogeneity as a rich source of information and to identify research gaps.[47]

Deliverable: Enriched understanding of the tool's usability, acceptability, and perceived effectiveness and relevance to key sociodemographic differences in order to optimize tailored communication of the tool.

Feasibility and mitigating strategies

Our existing relationships, COVID-19 research infrastructure, and active partnerships with national youth and family organizations, in collaboration with the Centre for Addiction and Mental Health and National Youth Advisory Council will enable us to facilitate timely uptake and dissemination of our results. The primary limitations of this work are loss to follow-up and low eHealth literacy that would threaten the internal validity of our findings through selection bias. Our partnerships with community-based and health system networks will help to mitigate this issue. In all phases of work we will provide clear verbal instructions and a slowed pace of speech that uses plain and avoids jargon. Missing data may also present a threat to our study through selection bias. We will pilot materials with youth and family partners on our team to ensure the length is manageable (less than. 20 minutes to complete). Finally, it is important to recognize that the COVID-19 pandemic and associated public health measures are not universally detrimental to youth; they may have experienced a reduction in school-life conflict[48] or in stress from potential high-risk COVID-19 exposures in their classrooms.[49 50] Our deliverables will be relevant to youth experiencing mental health difficulties in the COVID-19 recovery period, and beyond.

For peer review only - http://bmjopen.bmj.com/site/about/guidelines.xhtml

Funding Statement

Dr. Parsons Leigh obtained funding for this work from the Canadian Institutes of Health Research (CIHR) (#483889). Dr. Moss was supported by a CIHR Banting Postdoctoral Fellowship. CIHR has no role in the study design; data collection, management, analysis, or interpretation; writing of this or future reports; the decision to submit for publication; or ultimate authority over any of these activities.

Author Contributions Statement

SJM, NR, HTS, and JPL conceptualized the study. HTS, KMF, and JPL provided resources for the conduction of this work, while JPL provided the software. NR, HTS, KMF, and JPL have supervised the production of this protocol and will supervise the conduction of the work. SJM, MSC, and JPL wrote the original draft of this protocol and all authors reviewed and edited this protocol prior to submission for peer review.

Data Sharing and Availability of Materials

Not applicable. No data was analyzed for development of this protocol.

Competing interests

The authors declare that they have no competing interests relevant to this work.

Acknowledgements

The authors wish to acknowledge their organizational partners including Solutions for Kids in Pain, the O'Brien Institute for Public Health, the Maritime and Alberta SPOR SUPPORT Units, 19 to Zero, The Sandbox Project, Children's Healthcare Canada, the Centre for Addictions and Mental Health, the Centre for Child, Youth, and Family Mental Health, and the Young Canadians Roundtable on Mental Health. The authors thank the youth and family dyads who are engaged and invested in bettering tomorrow for others.

For peer review only

For peer review only - http://bmjopen.bmj.com/site/about/guidelines.xhtml

Bibiliography

1. Marques de Miranda D, da Silva Athanasio B, Sena Oliveira AC, et al. How is COVID-19 pandemic impacting mental health of children and adolescents? *International Journal of Disaster Risk Reduction* 2020;51:101845. doi: https://doi.org/10.1016/j.ijdrr.2020.101845
2. Silliman Cohen RI, Bosk EA. Vulnerable Youth and the COVID-19 Pandemic. *Pediatrics* 2020;146(1):e20201306. doi: 10.1542/peds.2020-1306
3. Ramalho R, Adiukwu F, Gashi Bytyçi D, et al. Telepsychiatry and healthcare access inequities during the COVID-19 pandemic. *Asian Journal of Psychiatry* 2020;53:102234. doi: https://doi.org/10.1016/j.ajp.2020.102234
4. Slattery P, Saeri AK, Bragge P. Research co-design in health: a rapid overview of reviews. *Health Research Policy and Systems* 2020;18(1):17. doi: 10.1186/s12961-020-0528-9
5. Eyles H, Jull A, Dobson R, et al. Co-design of mHealth Delivered Interventions: A Systematic Review to Assess Key Methods and Processes. *Current Nutrition Reports* 2016;5(3):160-67. doi: 10.1007/s13668-016-0165-7
6. Pellicano E, Stears M. The hidden inequalities of COVID-19. *Autism* 2020;24(6):1309-10. doi: 10.1177/1362361320927590
7. Power E, Hughes S, Cotter D, et al. Youth mental health in the time of COVID-19. *Irish Journal of Psychological Medicine* 2020;37(4):301-05. doi: 10.1017/ipm.2020.84 [published Online First: 2020/07/02]
8. Rauschenberg C, Schick A, Goetzl C, et al. Social isolation, mental health, and use of digital interventions in youth during the COVID-19 pandemic: A nationally representative survey. *European Psychiatry* 2021;64(1):e20. doi: 10.1192/j.eurpsy.2021.17 [published Online First: 2021/03/09]
9. Jiang X, Ming W-K, You JHS. The Cost-Effectiveness of Digital Health Interventions on the Management of Cardiovascular Diseases: Systematic Review. *J Med Internet Res* 2019;21(6):e13166. doi: 10.2196/13166
10. Murray E, Hekler EB, Andersson G, et al. Evaluating Digital Health Interventions: Key Questions and Approaches. *Am J Prev Med* 2016;51(5):843-51. doi: 10.1016/j.amepre.2016.06.008
11. Pretorius C, Coyle D. Young People's Use of Digital Tools to Support Their Mental Health During Covid-19 Restrictions. *Frontiers in Digital Health* 2021;3 doi: 10.3389/fdgth.2021.763876
12. Rauschenberg C, Schick A, Hirjak D, et al. Evidence Synthesis of Digital Interventions to Mitigate the Negative Impact of the COVID-19 Pandemic on Public Mental Health: Rapid Meta-review. *J Med Internet Res* 2021;23(3):e23365. doi: 10.2196/23365
13. Malboeuf-Hurtubise C, Léger-Goodes T, Mageau GA, et al. Philosophy for children and mindfulness during COVID-19: Results from a randomized cluster trial and impact on mental health in elementary school students. *Progress in Neuro-Psychopharmacology and Biological Psychiatry* 2021;107:110260. doi: https://doi.org/10.1016/j.pnpbp.2021.110260
14. Roberts LW, Chan S, Torous J. New tests, new tools: mobile and connected technologies in advancing psychiatric diagnosis. *NPJ Digit Med* 2018;1:20176. doi: 10.1038/s41746-017-0006-0 [published Online First: 20180226]
15. Clarke AM, Kuosmanen T, Barry MM. A systematic review of online youth mental health promotion and prevention interventions. *Journal of youth and adolescence* 2015;44:90-113.
16. Bäuml J, Froböse T, Kraemer S, et al. Psychoeducation: a basic psychotherapeutic intervention for patients with schizophrenia and their families. *Schizophr Bull* 2006;32 Suppl 1(Suppl 1):S1-S9. doi: 10.1093/schbul/sbl017 [published Online First: 2006/08/18]
17. Lincoln TM, Wilhelm K, Nestoriuc Y. Effectiveness of psychoeducation for relapse, symptoms, knowledge, adherence and functioning in psychotic disorders: A meta-analysis. *Schizophrenia Research* 2007;96(1):232-45. doi: https://doi.org/10.1016/j.schres.2007.07.022
18. Asmundson GJG, Blackstock C, Bourque MC, et al. Easing the disruption of COVID-19: supporting the mental health of the people of Canada—October 2020—an RSC Policy Briefing. *FACETS* 2020;5(1):1071-98. doi: 10.1139/facets-2020-0082

For peer review only - http://bmjopen.bmj.com/site/about/guidelines.xhtml

19. Seabury SA, Axeen S, Pauley G, et al. Measuring The Lifetime Costs Of Serious Mental Illness And The Mitigating Effects Of Educational Attainment. *Health Aff (Millwood)* 2019;38(4):652-59. doi: 10.1377/hlthaff.2018.05246
20. Gill M, Bagshaw SM, McKenzie E, et al. Patient and Family Member-Led Research in the Intensive Care Unit: A Novel Approach to Patient-Centered Research. *PLoS One* 2016;11(8):e0160947. doi: 10.1371/journal.pone.0160947 [published Online First: 2016/08/06]
21. Krewulak KD, Sept BG, Stelfox HT, et al. Feasibility and acceptability of family administration of delirium detection tools in the intensive care unit: a patient-oriented pilot study. *CMAJ Open* 2019;7(2):E294-e99. doi: 10.9778/cmajo.20180123 [published Online First: 2019/04/28]
22. Farrier CE, Stelfox HT, Fiest KM. In the pursuit of partnership: patient and family engagement in critical care medicine. *Curr Opin Crit Care* 2019;25(5):505-10. doi: 10.1097/mcc.0000000000000643 [published Online First: 2019/07/18]
23. de Grood C, Leigh JP, Bagshaw SM, et al. Patient, family and provider experiences with transfers from intensive care unit to hospital ward: a multicentre qualitative study. *CMAJ* 2018;190(22):E669-E76.
24. Ross DA, Hinton R, Melles-Brewer M, et al. Adolescent Well-Being: A Definition and Conceptual Framework. *J Adolesc Health* 2020;67(4):472-76. doi: 10.1016/j.jadohealth.2020.06.042 [published Online First: 2020/08/18]
25. Ross DA, Hinton R, Melles-Brewer M, et al. Adolescent Well-Being: A Definition and Conceptual Framework. *The Journal of adolescent health : official publication of the Society for Adolescent Medicine* 2020;67(4):472-76. doi: 10.1016/j.jadohealth.2020.06.042 [published Online First: 2020/08/13]
26. Schoonenboom J, Johnson RB. How to Construct a Mixed Methods Research Design. *Kolner Z Soz Sozpsychol* 2017;69(Suppl 2):107-31.
27. Fetters MD, Curry LA, Creswell JW. Achieving integration in mixed methods designs-principles and practices. *Health Serv Res* 2013;48(6 Pt 2):2134-56. doi: 10.1111/1475-6773.12117 [published Online First: 20131023]
28. Johnson RB, Onwuegbuzie AJ. Mixed methods research: A research paradigm whose time has come. *Educational researcher* 2004;33(7):14-26.
29. Johnson RB, Onwuegbuzie AJ, Turner LA. Toward a definition of mixed methods research. *Journal of mixed methods research* 2007;1(2):112-33.
30. Fiest KM, McIntosh CJ, Demiantschuk D, et al. Translating evidence to patient care through caregivers: a systematic review of caregiver-mediated interventions. *BMC Med* 2018;16(1):105. doi: 10.1186/s12916-018-1097-4
31. Government of Canada. Top 5 languages spoken in Canada Ottawa, ON2018 [updated Sept 18, 2020. Available from: https://www.clo-ocol.gc.ca/en/newsletter/2018/top-5-languages-spoken-canada accessed April 8 2021.
32. Thompson V. Why are youth flocking to TikTok to cope with the COVID-19 pandemic? 2020. https://news.yorku.ca/2020/04/02/why-are-youth-flocking-to-tiktok-to-cope-with-the-covid-19-pandemic/.
33. Bryson A. In age of coronavirus, could social media be kids' saving grace? *Desert Sun* 2020 April 12, 2020. https://www.desertsun.com/story/news/education/2020/04/12/coronavirus-kids-cope-pandemic-snapchat-tiktok-instagram/5126302002/ (accessed July 22, 2021).
34. Joanna Briggs Institute. Checklist for Qualitative Research: Joanna Briggs Institute; 2017 [Available from: https://jbi.global/sites/default/files/2019-05/JBI_Critical_Appraisal-Checklist_for_Qualitative_Research2017_0.pdf accessed March 23 2020.
35. Brouwers MC, Kerkvliet K, Spithoff K. The AGREE Reporting Checklist: a tool to improve reporting of clinical practice guidelines. *BMJ* 2016;352:i1152. doi: 10.1136/bmj.i1152
36. Morgan D, Krueger R, King J. Focus group kit. Thousand Oaks, CA: SAGE Publications 1998.
37. Parsons M, Greenwood J. A guide to the use of focus groups in health care research: Part 1. *Contemp Nurse* 2000;9(2):169-80. doi: 10.5172/conu.2000.9.2.169 [published Online First: 2002/02/22]
38. Shoemaker SJ, Wolf MS, Brach C. The Patient Education Materials Assessment Tool (PEMAT) and User's Guide Rockville, MD: Agency for Healthcare Research and Quality; [updated November 2020. Available from: https://www.ahrq.gov/health-literacy/patient-education/pemat.html.

For peer review only

39. Nielsen J. Usability engineering. Boston: Academic Press 1993.
40. Schorr C, Hunter K, Zuzelo PR. Understandability and Actionability of the CDC'S Printable Sepsis Patient Education Material. *Am J Crit Care* 2018;27(5):418-27. doi: 10.4037/ajcc2018121 [published Online First: 2018/09/03]
41. Sandelowski M, Barroso J, Voils CI. Using qualitative metasummary to synthesize qualitative and quantitative descriptive findings. *Res Nurs Health* 2007;30(1):99-111. doi: 10.1002/nur.20176
42. Topp CW, Østergaard SD, Søndergaard S, et al. The WHO-5 Well-Being Index: a systematic review of the literature. *Psychother Psychosom* 2015;84(3):167-76. doi: 10.1159/000376585 [published Online First: 20150328]
43. Smith JG, Alamiri NS, Biegger G, et al. Think-Aloud Testing of a Novel Safer Drinking App for College Students During COVID-19: Usability Study. *JMIR Form Res* 2022;6(2):e32716. doi: 10.2196/32716 [published Online First: 20220217]
44. Giang WCW, Bland E, Chen J, et al. User Interactions With Health Insurance Decision Aids: User Study With Retrospective Think-Aloud Interviews. *JMIR Hum Factors* 2021;8(4):e27628. doi: 10.2196/27628 [published Online First: 20211026]
45. Busse TS, Jux C, Kernebeck S, et al. Participatory Design of an Electronic Cross-Facility Health Record (ECHR) System for Pediatric Palliative Care: A Think-Aloud Study. *Children (Basel)* 2021;8(10) doi: 10.3390/children8100839 [published Online First: 20210924]
46. Bangor A, Kortum PT, Miller JT. An Empirical Evaluation of the System Usability Scale. *International Journal of Human–Computer Interaction* 2008;24(6):574-94. doi: 10.1080/10447310802205776
47. Petticrew M, Rehfuess E, Noyes J, et al. Synthesizing evidence on complex interventions: how meta-analytical, qualitative, and mixed-method approaches can contribute. *J Clin Epidemiol* 2013;66(11):1230-43. doi: 10.1016/j.jclinepi.2013.06.005 [published Online First: 2013/08/21]
48. Graham M, Weale V, Lambert KA, et al. Working at Home: The Impacts of COVID 19 on Health, Family-Work-Life Conflict, Gender, and Parental Responsibilities. *J Occup Environ Med* 2021;63(11):938-43. doi: 10.1097/jom.0000000000002337
49. Schwartz KD, Exner-Cortens D, McMorris CA, et al. COVID-19 and Student Well-Being: Stress and Mental Health during Return-to-School. *Canadian journal of school psychology* 2021;36(2):166-85. doi: https://dx.doi.org/10.1177/08295735211001653
50. Lan F-Y, Suharlim C, Kales SN, et al. Association between SARS-CoV-2 infection, exposure risk and mental health among a cohort of essential retail workers in the USA. *Occupational and Environmental Medicine* 2021;78(4):237. doi: 10.1136/oemed-2020-106774

For peer review only
