# Supplementary material for: Codesigning a user-centred digital psychoeducational tool for youth mental well-being with families in Canada: study protocol for a sequential exploratory mixed methods study
Source: BMJ Open. 2023 Jun 26;13(6):e072533. doi: 10.1136/bmjopen-2023-072533 (PMC10410808; doi:10.1136/bmjopen-2023-072533)
Supplement: Reviewer comments [file bmjopen-2023-072533.reviewer_comments.pdf]

PEER REVIEW HISTORY

BMJ Open publishes all reviews undertaken for accepted manuscripts. Reviewers are asked to complete a checklist review form (**http://bmjopen.bmj.com/site/about/resources/checklist.pdf**) and are provided with free text boxes to elaborate on their assessment. These free text comments are reproduced below.

ARTICLE DETAILS

**TITLE (PROVISIONAL)**	Co-designing a User-centered Digital Psychoeducational Tool for Youth Mental Wellbeing with Families in Canada: Study Protocol for a Sequential Exploratory Mixed Methods Study
**AUTHORS**	Moss, Stephana; Racine, Nicole; Ahmed, Sofia; Birnie, kathryn; Cherak, Michal S; Curran, Janet; Halperin, Donna; Halperin, Scott; Harley, Micaela; Hu, Jia; Leppan, Laura; Nickel, Angie; Russell, Kristine; Solis, May; Smith, Stacie; Soo, Andrea; Stelfox, Maia; Tutelman, Perri; Stelfox, Henry; Fiest, Kirsten; Parsons Leigh, Jeanna

This article was not externally reviewed at BMJ Open. Protocols that have been independently assessed prior to submission to BMJ Open are usually fast-tracked to publication on the grounds that further substantial changes will not be possible. This independent assessment will usually be external, independent review for both a substantial grant award from a non-commercial or government funder and ethics approval.
